# Comparative Analysis of MAR and Terminator Elements in Controlling Stable EPO Expression in CHO-K1 Cells

**DOI:** 10.34172/apb.45955

**Published:** 2026-06-27

**Authors:** Fatemeh Jalali, Shahriyar Abdoli, Fatemeh Shamsabadi, Ahad Yamchi, Anvarsadat Kianmehr, Majid Shahbazi

**Affiliations:** ^1^Medical Cellular and Molecular Research Center, Golestan University of Medical Sciences, Gorgan, Iran; ^2^Department of Medical Biotechnology, Faculty of Advanced Medical Technologies, Golestan University of Medical Sciences, Gorgan, Iran; ^3^Department of Plant Breeding and Biotechnology, College of Plant Production, Gorgan University of Agricultural Sciences and Natural Resources, Gorgan, Iran; ^4^Arya Tina Gene (ATG) Biopharmaceutical Company, Gorgan, Iran

**Keywords:** MAR, Terminator, Erythropoietin, CHO K-1, Stable cell line

## Abstract

**Purpose:**

Chromosome remodeling, mRNA 3’ end extension diversity, mRNA maturation, and the utilization of upstream and downstream DNA elements have been shown to significantly impact the production of recombinant proteins. In this study, we employed the human beta-globin Matrix Attachment Region (β-MAR) as an upstream element and the human beta-globin terminator (β-TERM) as a downstream section to augment erythropoietin (EPO) production.

**Methods:**

Four plasmids expressing the EPO transgene, after optimization for codon bias and secondary structure of the EPO mRNA, were transfected into CHO-K1 cells. Two weeks after antibiotic selection, pooled stable clones were evaluated for gene copy number (GCN), mRNA quantification, and EPO expression level.

**Results:**

Our results revealed a significant increase in the amount of EPO secreted by stable CHO cells in the presence of β-MAR. At the same time, β-TERM had no noticeable impact on EPO production. As expected, the presence of the β-MAR sequence resulted in a higher gene copy number with 15 gene copy per cell (GCN/cell) and mRNA quantity with 5.3 fold change, leading to a much greater secretion of EPO with 28.3 mIU/ml compared to the β-TERM sequence with 3 GCN/cell, 2.8 mRNA fold change and 8.2 mIU/ml of EPO quantity. In the other construct incorporating both β-MAR and β-TERM elements, a gene copy number (GCN) of 7.0 per cell was detected, accompanied by a 4.58-fold elevation in mRNA expression and a secreted erythropoietin (EPO) concentration of 22.45 mIU/ml. In contrast, the construct devoid of both β-MAR and β-TERM (used as the calibrator) elements demonstrated a GCN of 1.0 per cell, an mRNA expression fold change of 1.0, and an EPO concentration of 8.35 mIU/ml.

**Conclusion:**

This experiment further supports previous studies, demonstrating the significant impact of the β-MAR element on recombinant protein production in stable CHO-K1 cells.

## Introduction

 Cell engineering has emerged as a powerful strategy for optimizing host systems to produce recombinant proteins with structural and functional properties comparable to those synthesized in native eukaryotic cells.^1^ The growing demand for complex biopharmaceutical products, including glycoproteins and hormones, has accelerated the development of engineered cellular platforms capable of supporting proper protein folding, post-translational modifications, and biological activity.^2^ Although microbial hosts such as *Escherichia coli* and yeast offer advantages in terms of simplicity and scalability, their inherent limitations in processing complex eukaryotic proteins necessitated the use of mammalian expression systems.

 Among mammalian hosts, Chinese hamster ovary (CHO) cells remain the most widely used platform for therapeutic protein production due to their ability to perform human-compatible post-translational modifications.^3,4^ Nevertheless, recombinant protein expression in CHO cells is often constrained by position-dependent integration effects, transcriptional silencing, heterogeneous expression levels, and limited long-term stability.^1^ These challenges underscore the need for advanced cell engineering strategies to improve expression efficiency and product consistency.

 Multiple approaches have been developed to enhance recombinant protein production in mammalian systems, including host genome engineering, vector optimization, epigenetic modulation, and post-transcriptional regulation.^3,5^ Among these, the incorporation of regulatory DNA elements into expression constructs has attracted considerable attention. In particular, engineering of untranslated regions (UTRs) represents an effective strategy for modulating gene expression at both transcriptional and post-transcriptional levels.^6^

 Matrix Attachment Regions (MARs) are cis-acting DNA elements that anchor chromatin to the nuclear matrix, thereby establishing transcriptionally active domains and reducing position-effect variegation.^7-9^ MARs contain binding sites for transcription factors and structural proteins, enabling coordinated regulation of chromatin architecture and gene expression. Several studies have demonstrated that MARs can reduce gene silencing and promote prolonged and stable transgene expression in mammalian cells, although their functional performance may vary depending on vector architecture, promoter context, and genomic integration environment.^10-12^

 In parallel, transcriptional terminator (TERM) sequences play a crucial role in accurate transcription termination, prevention of transcriptional read-through, and stabilization of mRNA transcripts.^13,14^ The composition and length of the 3′ untranslated region (3′-UTR) influence mRNA stability, processing efficiency, and regulatory interactions such as microRNA binding.^15,16^ β-globin terminator elements (β-TERM, γ-TERM) have been shown to outperform commonly used polyadenylation signals, such as SV40 polyA, in suppressing transcriptional read-through and sustaining long-term gene expression in stable mammalian systems.^17^ Moreover, β-globin-derived 3′-UTRs have gained recognition as gold-standard regulatory elements in mRNA vaccine platforms due to their ability to enhance mRNA stability and protein output.^18,19^

 Targeting chromatin-regulating and transcription termination elements to distinct regions of expression cassettes provides a rational framework for coordinated regulation of transcriptional activity, RNA processing, and transgene stability. Although the individual effects of MARs and β-globin terminator elements on recombinant gene expression have been investigated in mammalian systems, most previous studies have focused on single regulatory elements or distinct vector configurations.^7,11,20^ Consequently, the functional compatibility and combined regulatory effects of β-MAR and β-TERM within a unified mammalian expression cassette remain insufficiently characterized, particularly in the context of stable erythropoietin (EPO) production in CHO cells.

 In this study, we performed a systematic comparative evaluation of β-MAR and β-TERM regulatory elements, individually and in combination, within a single codon-optimized EPO expression cassette in stable CHO-K1 cell pools. By analyzing transgene copy number, steady-state mRNA abundance, and secreted EPO production across four defined vector architectures, we aimed to investigate how chromatin-associated and post-transcriptional regulatory elements collectively influence recombinant protein expression. Rather than proposing a fundamentally novel regulatory mechanism, this study was designed to provide a comparative framework for optimizing expression cassette architecture and supporting rational vector design in mammalian cell engineering.

## Materials and Methods:

###  Design and Assembly of EPO Expression Construct

 The nucleotide sequences of human erythropoietin (EPO; NM_000799.4, nucleotides 514–1095), the human β-globin matrix attachment region (MAR; GenBank accession no. L22754.1), and the human β-globin transcription terminator sequence (β-TERM; GenBank accession no. U01317) were retrieved from the NCBI database.

 The EPO coding sequence was codon-optimized to enhance expression efficiency in CHO-K1 cells by accounting for codon usage bias and mRNA secondary structure, as described by Yamchi et al.^21^ Codon optimization was evaluated using multiple parameters, including the codon adaptation index (CAI), codon frequency units (CFU), codon pair frequency units, GC content, and the presence of cis-negative elements within the coding sequence. These analyses were performed using the CHO codon usage database available through the Kazusa Codon Usage Database (http://www.kazusa.or.jp/codon/).

 In addition, the secondary structure of the EPO mRNA was optimized across the entire coding sequence. The stability of predicted RNA secondary structures, including hairpin formations, was assessed using the RNAfold web server from the ViennaRNA package (http://rna.tbi.univie.ac.at/cgi-bin/RNAWebSuite/RNAfold.cgi).

 All optimized and regulatory sequences were synthesized commercially (BioBasic, Canada) and subsequently subcloned into the PiggyBac expression vector (PB513B-1) at the appropriate multiple cloning sites. This vector contains a cytomegalovirus (CMV) promoter and a human β-globin polyadenylation signal (polyA). Standard molecular cloning and expression techniques were employed for vector construction, as illustrated in Figure 1.

**Figure 1:**
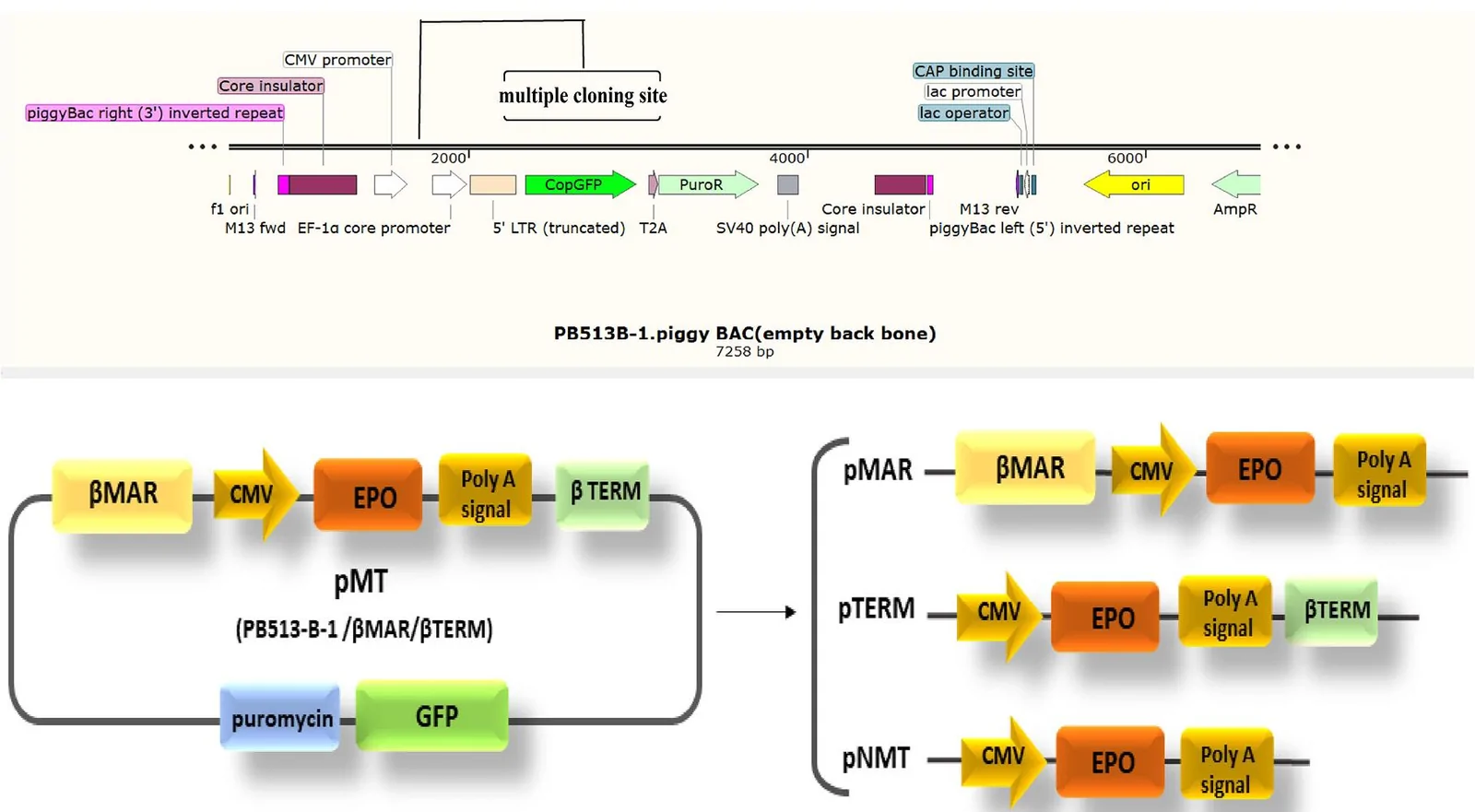
Schematic representation of four PiggyBac-based expression plasmids generated by digestion and ligation. The constructs include pMT (containing both β-MAR and β-TERM sequences), pMAR (containing β-MAR only), pTERM (containing β-TERM only), and pNMT (lacking both β-MAR and β-TERM and used as the control). All vectors were designed, subcloned, linearized, and transfected into CHO-K1 cells

###  In Silico Prediction and Network Analysis of RNA-Binding Proteins Associated with the β-TERM

 To predict RNA-binding proteins (RBPs) potentially interacting with the β-TERM sequence, an *in silico* analysis was performed using the RBPmap web server (https://rbpmap.technion.ac.il/). RBPs predicted to bind both the polyA signal and β-TERM sequence were subsequently identified and selected for downstream protein-protein interaction (PPI) analysis using the STRING database (https://string-db.org/). Network visualization and hub protein identification were conducted using the Cytoscape software. Functional enrichment analyses, including molecular function and pathway annotations, were performed using the PANTHER classification system (https://pantherdb.org/).

###  Cell Culture and Transfection

 CHO-K1 cells were obtained from the Pasteur Institute of Iran and cultured in DMEM/F12 medium supplemented with 10% fetal bovine serum (FBS; Gibco, Thermo Fisher Scientific) in a humidified incubator at 37 °C with 5% CO₂. Actively growing cells were seeded into 24-well plates at appropriate densities before transfection.

 All expression plasmids were digested and linearized at the ampicillin resistance gene using FastDigest ScaI restriction enzyme (#FD0434, Thermo Fisher Scientific). Twenty-four hours after seeding, cells were transfected with the linearized plasmids using ScreenFect transfection reagent (FUJIFILM, Japan), according to the manufacturer’s instructions.

###  Establishment of Stable Recombinant CHO-K1 Cell Lines

 To establish stable CHO-K1 cell lines expressing recombinant EPO constructs, puromycin selection was initiated 48 h post-transfection by replacing the culture medium with selection medium containing 4 μg/mL puromycin (BioBasic, Canada). The optimal puromycin concentration was determined in advance using a minimum inhibitory concentration (MIC) assay. Selection medium was refreshed every 2–3 days until puromycin-resistant clones became visible.

 Well-established resistant clones were subsequently expanded by transferring them from 24-well plates to T-25 flasks containing the same selection medium. After two weeks of continuous selection, culture supernatants from stable pooled cell populations were collected and stored at −70 °C for downstream analyses. In parallel, stable pooled cells were cryopreserved in liquid nitrogen for long-term storage.

###  Quantification of EPO Gene Copy Number

 Absolute quantification of EPO gene copy number (GCN) was performed by quantitative PCR (qPCR) using a StepOnePlus^TM^ Real-Time PCR System (Applied Biosystems, USA). Reactions were carried out using RealQ Plus 2 × Master Mix Green with High ROX^TM^ (Ampliqon, Denmark) and EPO-specific primers (Table 1).

**Table 1:** Primer sequences used for quantitative analysis of EPO GCN and transcripti

**Gene name**	**Sequences**	**Amplicon size (bp)**
Erythropoietin	F: TGCTGTTTGCCCTGATCTG R: CACGGTGATGTTCTCGTTCAG	130
CHO Beta Actin	F: GCCTTCCTTCCTGGGTATG R: CTTGATCTTCATGGTGCTGG	196

 A standard curve was generated using 10-fold serial dilutions of the pMAR expression plasmid, covering a range from 8.7 × 10⁹ to 87 × 10^2^ copies per reaction. Plasmid copy numbers were calculated using an online copy number calculator (https://scienceprimer.com/copy-number-calculator-for-realtime-pcr).

 Genomic DNA (gDNA) was isolated from 2 × 10⁶ cells of each stable CHO-K1 pool using a genomic DNA purification kit (SinaClon, Iran). For each reaction, 20 ng of gDNA was used as template. Amplification was performed with an initial denaturation step at 95 °C for 15 min, followed by 35 cycles of denaturation at 95 °C for 15 s and annealing/extension at 61 °C for 40 s. Amplification specificity was verified by melting curve analysis.

 GCN analysis was performed using three independent biological replicates, with each sample analyzed in technical triplicate.

 Absolute EPO copy numbers were determined by interpolation of Ct values against the plasmid-derived standard curve according to Equation 1:


(1) $$Ct=\;\left(slope.\;log\;GCN\right)+c$$

Where *c* represents the y-intercept of the standard curve (R^2^ = 0.98).

 To determine the number of EPO copies per cell, the absolute copy number obtained from the standard curve was normalized to the amount of gDNA analyzed. The DNA content of a diploid CHO-K1 cell was assumed to be 5.4 pg.^22^ Since 20 ng (20000 pg) of gDNA was used in each reaction, the EPO GCN per cell was calculated according to Equation 2:


(2) $$genecopy\;per\;cell:\;\frac{\left(GCN\right)\;5.4}{20000}$$

Where Absolute GCN represents the total number of EPO copies detected in the qPCR reaction.

###  Transcriptional Analysis of Recombinant EPO Expression

 EPO mRNA levels in each stable cell pool were quantified by quantitative real-time PCR (qRT-PCR) using a StepOnePlus^TM^ Real-Time PCR System (Applied Biosystems, USA) with RealQ Plus 2x Master Mix Green containing high ROX^TM^ (Ampliqon, Denmark).

 Total RNA was manually isolated from 1 × 10⁶ cells at the mid-exponential growth phase using RNX Plus reagent (SinaClon, Iran). RNA concentration and purity were assessed using a NanoDrop 1000 spectrophotometer, and samples with an A₂₆₀/A₂₈₀ ratio of approximately 2.0 were considered suitable for downstream applications. Up to 1 µg of total RNA was reverse-transcribed into cDNA using a commercial cDNA synthesis kit (FAVORGEN, Taiwan). The resulting cDNA was diluted 1:10 with nuclease-free water.

 qRT-PCR amplification was performed using 200 ng of diluted cDNA per reaction under the following thermal cycling conditions: an initial denaturation at 95 °C for 15 min, followed by 35 cycles of denaturation at 95 °C for 15 s and annealing/extension at 60 °C for 30 s. Reaction specificity was confirmed by melting curve analysis. Total RNA samples were obtained from three independent biological replicates. Each qRT-PCR reaction was performed in technical triplicate to ensure measurement accuracy.

 Relative EPO mRNA expression levels were calculated using the ΔΔCt method, with CHO β-actin serving as the endogenous reference gene for normalization. Primer sequences are listed in Table 1.

###  Assessment of EPO Protein Expression in Stable CHO-K1 Cells

 To assess EPO protein expression levels in stable CHO-K1 cell pools, conditioned culture media were analyzed by SDS-PAGE and Western blotting. Equal volumes (80 μL) of conditioned media from each stable pooled sample were subjected to electrophoresis on 12% Bis-Tris SDS-PAGE gels using a Mini-PROTEAN II system (Bio-Rad, USA). Before electrophoresis, samples were mixed with loading buffer containing 2-mercaptoethanol and heated at 100 °C for 5 min.

 Following protein separation, Western blotting was performed. Proteins were transferred onto a nitrocellulose membrane using a wet transfer system (Bio-Rad, USA) at 100V for 90 min. The membrane was blocked with 3% (w/v) skim milk in phosphate-buffered saline containing 0.1% Tween-20 (PBST) for 2 h at room temperature, followed by three washes with PBST.

 The membrane was then incubated overnight at 4 °C with a rabbit anti-EPO primary antibody diluted 1:5000 (Abcam, UK). After three washes with PBST, the membrane was incubated for 2 h at room temperature with horseradish peroxidase (HRP)-conjugated goat anti-rabbit IgG secondary antibody diluted 1:1000 (Sigma-Aldrich, Germany). The membrane was subsequently washed three times with PBST, and immunoreactive bands were visualized using 3,3′-diaminobenzidine tetrahydrochloride (DAB) substrate (Sigma-Aldrich, Germany) for 3–5 min. Western blot analyses were conducted using protein extracts obtained from three independent biological replicates to confirm reproducibility of EPO expression patterns.

###  Quantification of Secreted EPO by ELISA

 To quantify secreted EPO, 5 × 10⁵ cells from each stable cell pool were seeded into T-25 flasks. After 48 h of incubation, conditioned culture medium was collected. Prior to analysis, all samples were diluted to a final dilution factor of 1:10,000 using the assay diluent to ensure measurements within the linear range of the standard curve. EPO concentrations were determined using the LEGEND MAX^TM^ Human Erythropoietin ELISA Kit (Cat. No. 442907, BioLegend, USA), according to the manufacturer’s instructions. Quantification was performed using samples from three independent biological experiments, with each sample measured in technical triplicate

###  Statistical Analysis

 Statistical analyses were performed using GraphPad Prism version 8 (GraphPad Software, USA). Differences among experimental groups were evaluated using one-way analysis of variance (ANOVA) followed by Tukey’s post hoc test for multiple pairwise comparisons. A *P* value < 0.05 was considered statistically significant whereas, the lesser P value only strengthened the significance. All quantitative data are presented as mean ± standard deviation (SD) derived from three independent biological experiments.

## Results

###  Codon Optimization of EPO mRNA 

 Codon optimization of the EPO coding sequence resulted in a CAI of 0.99 and a Transfer RNA Adaptation Index (tAI) of 0.98, indicating high compatibility with the translational machinery of CHO cells. Following optimization, the average GC content of the EPO gene increased to 67.7%. In addition, several predicted negative cis-acting elements within the coding sequence, including RNase-sensitive motifs, destabilizing elements, cryptic splice sites, and premature transcription termination signals, were eliminated.

###  RNA Secondary Structure Analysis of EPO–PolyA and EPO–PolyA–β-TERM Transcripts

 RNA secondary structure prediction using RNAfold revealed changes in the thermodynamic profile of EPO mRNA following codon optimization. Specifically, the minimum free energy (MFE) of the EPO transcript shifted from −273.30 kcal/mol to −263.10 kcal/mol.

 Secondary structure analysis of extended transcripts revealed that the inclusion of downstream regulatory elements substantially altered overall RNA folding energetics. The predicted MFE of the EPO–PolyA transcript was −358.60 kcal/mol, whereas incorporation of both the β-globin polyadenylation signal and the β-globin transcription terminator (EPO–PolyA–β-TERM) resulted in a further decrease in MFE to −586.10 kcal/mol (Figure 2a). For both constructs, MFE and centroid structures were generated to visualize RNA secondary conformations and assess global folding patterns (Figure 2b).

**Figure 2:**
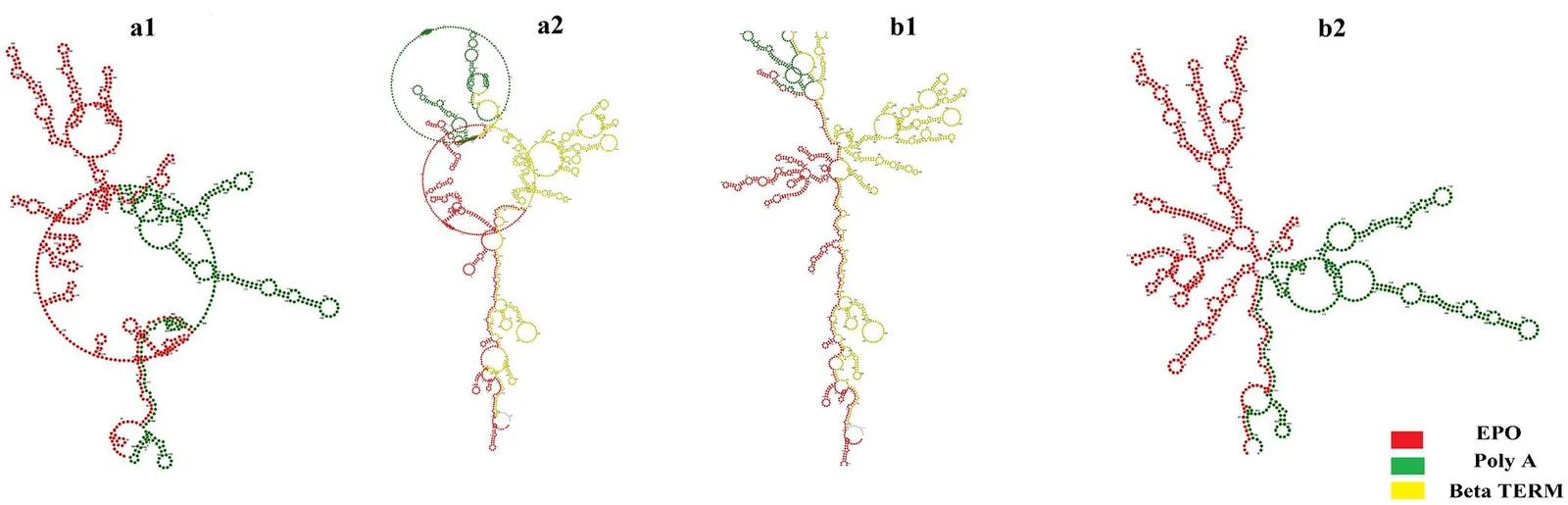
RNA secondary structure prediction of EPO mRNA constructs using RNAfold. Predicted RNA secondary structures of EPO transcripts generated using the RNAfold web server. (a1, a2) MFE and centroid structures are shown for EPO alone and (b1, b2) EPO–PolyA–TERM constructs. The EPO coding sequence is highlighted in red, the human β-globin polyadenylation signal in green, and the β-globin transcription terminator in yellow. Structures are displayed according to their predicted thermodynamic folding, with corresponding MFE values indicated for each construct

###  In Silico Identification of RBPs Interacting with the β-TERM

 RBPmap analysis identified a broad spectrum of predicted RBPs that interact with both the polyA signal and β-TERM (Table 2). Comparative assessment revealed a substantial overlap between the two elements, including members of the hnRNP family (HNRNPA0, HNRNPA1, HNRNPA2B1, HNRNPC, HNRNPK, and HNRNPD/DL), HuR (ELAVL proteins), PABPC1/4, PABPN1, CPEB1/2/4, TUT1, KHSRP, SFPQ, U2AF2, FXR1, IGF2BP2/3, PUM1/2, MSI1, and ZFP36. The presence of this shared RBP repertoire suggests that the β-TERM extends its function beyond transcription termination and engages regulatory mechanisms common to 3′ end processing and post-transcriptional control.

**Table 2:** Predicted RNA-binding proteins associated with the polyA signal and β-TERM

	**Predicted RNA-binding proteins**
**PolyA**	A1CF, BOLL, CELF1, CNOT4, CPEB1, CPEB2, CPEB4, DAZ3, DAZAP1, EIF4G2, ELAVL4, ENOX1, ESRP2, FUBP1, FUBP3, FUS, FXR1, HNRNPA0, HNRNPA1, HNRNPA1L2, HNRNPA2B1, HNRNPC, HNRNPCL1, HNRNPD, HNRNPDL, HNRNPF, HNRNPH1, HNRNPH2, HNRNPK, HNRNPL, HNRNPU, HNRPLL, HuR, IGF2BP2, IGF2BP3, KHDRBS1, KHDRBS2, KHDRBS3, KHSRP, MATR3, MSI1, NOVA1, NUPL2, PABPC1, PABPC3, PABPC4, PABPC5, PABPN1, PABPN1L, PCBP3, PCBP4, PRR3, PTB3, PTBP3, PUF60, PUM1, PUM2, QKI, RALY, RBFOX1, RBM15B, RBM24, RBM25, RBM38, RBM41, RBM42, RBM45, RBM46, RBM47, RBM5, RBM6, RBMS1, RBMS2, RBMS3, RC3H1, SART3, SF1, SFPQ, SNRNP70, SNRPA, SRSF10, SRSF11, SRSF2, SRSF5, SRSF8, TAF15, TIA1, TRA2A, TRNAU1AP, TUT1, U2AF2, YBX1, YBX2, ZC3H14, ZCRB1, ZFP36, ZNF326, ZNF638
**β-TERM**	**DAZAP1**, UNK, **YBX1**, **MSI1**, **HNRNPA0**, **SNRNP70**, **PRR3**, PCBP2, **PCBP3**, **RBM46**, **DAZ3**, G3BP2, **RBMS1**, **SFPQ**, **PCBP1**, **RBM41**, **RBM47**, **HuR**, **HNRNPA2B1**, **A1CF**, **KHSRP**, **ELAVL4**, **PUF60**, **RBMS2**, **RBMS3**, **TUT1**, **PCBP4**, **HNRNPK**, **CELF1**, **HNRNPA1**, **YBX2**, **RALY**, **CPEB2**, **U2AF2**, **TRA2A**, **RBM6**, **FUBP1**, **RBM24**, **BOLL**, **HNRNPH1**, **SART3**, **NUPL2**, **PABPC1**, **RC3H1**, **PABPC4**, **CPEB4**, TARDBP, **HNRNPC**, **SF1**, **HNRNPCL1**, FXR2, RBM23, FMR1, MBNL1, **RBM15B**, **NOVA1**, **TIA1**, **HNRNPDL**, **KHDRBS1**, **ZFP36**, **PABPN1**, **ZC3H14**, **ZCRB1**, RBM28, **KHDRBS3**, **HNRNPD**, **TRNAU1AP**, **PUM1**, **FUBP3**, **PUM2**, **PABPC3**, **PTBP3**, **KHDRBS2**, **QKI**, **PTB3**, **FXR1**, **RBM45**, **PABPN1L**, RBM3, **CPEB1**, HNRNPM, **ZNF326**, **RBFOX1**, SRSF1, RBM22, **MATR3**, SRSF9, **HNRNPA1L2**, **PABPC5**, **SRSF11**, **HNRNPU**, BRUNOL5, BRUNOL4, **HNRNPH2**, **SRSF2**, **HNRNPF**, **ZNF638**, **RBM42**, ANKHD1, **CNOT4**, **SNRPA**, RBM4B, LIN28A, **RBM25**, **RBM5**, **TAF15**, **IGF2BP2**, **SRSF10**, **FUS**, **SRSF5**, SRSF7, **IGF2BP3**, **ENOX1**, BRUNOL6

Note: Shared RNA-binding proteins are indicated in bold

###  Functional and Pathway Enrichment Analysis of Shared Poly(A)–β-TERM RBPs

 Functional enrichment analysis of RBPs shared between the polyA signal and β-TERM was performed using the PANTHER classification system. Protein class analysis revealed a strong enrichment of RNA metabolism–related proteins (PC00031), which constituted the majority of identified factors (64 proteins), including NOVA1, PTBP3, PUM2, SRSF5, PCBP3, CPEB4, and HNRNPA1L2. In addition, a subset of translational regulatory proteins (PC00263), such as FXR1, PABPN1/PABPN1L, TIA1, and ELAVL family members, was identified, indicating involvement in fine-tuning translational control. Furthermore, ZFP36 and SF1 were classified as gene-specific transcriptional regulators (PC00264).

 Pathway analysis demonstrated enrichment in transcription-related pathways, including general transcription regulation (P00023) and transcription regulation by bZIP transcription factors (P00055), predominantly driven by TAF15, as well as mRNA splicing pathways (P00058) represented by SNRPA. Consistently, molecular function analysis showed enrichment in transcription regulator activity (GO: 0140110; TAF15, ZFP36, and FUS) and translation regulator activity (GO: 0045182; BOLL, CPEB1/2/4, and DAZ3). These results indicate that the shared polyA–β-TERM–associated RBPs predominantly participate in post-transcriptional RNA processing and regulation rather than directly enhancing translational output.

###  Network-Based Identification of Key RBPs Associated with PolyA Signal and β-TERM

 Protein–protein interaction analysis of RBPs commonly predicted to bind both the polyA signal and the β-TERM revealed a densely interconnected network, indicating extensive functional coordination among post-transcriptional regulators (Figure 3a). Network construction using the STRING database revealed strong interactions among RBPs involved in RNA processing and stability.

**Figure 3:**
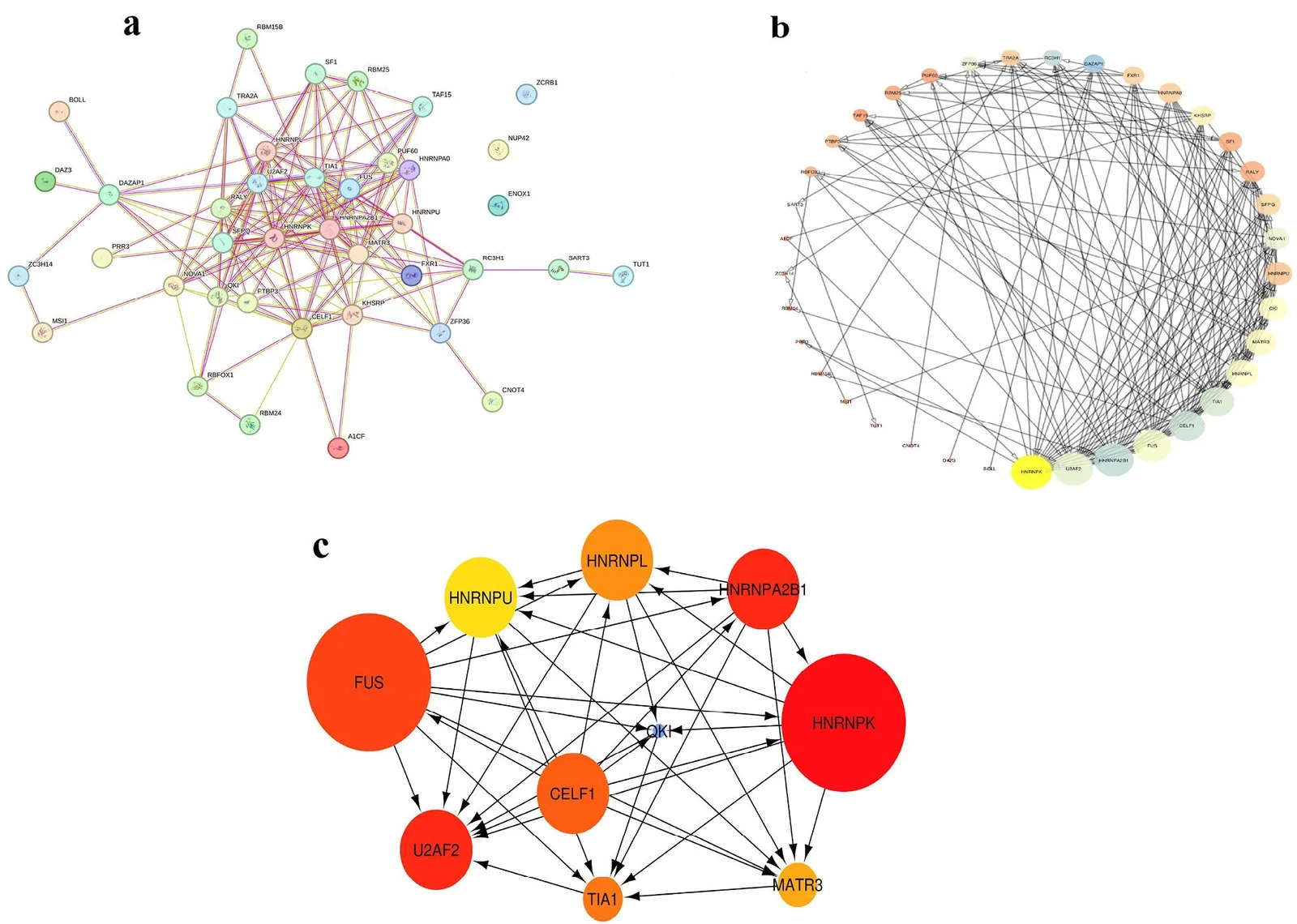
Protein–protein interaction network of RBPs associated with polyA signal and β-TERM. (a) The interaction network of RBPs common to both the polyA signal and β-TERM was constructed using the STRING database based on experimental evidence and text-mining criteria. (b) Network analysis was performed in Cytoscape using the CytoHubba plugin, ranking nodes according to degree and betweenness centrality. The thickness of the grey edges represents the strength of co-expression. HNRNPK, HNRNPA2B1, U2AF2, FUS, and CELF1 were identified as hub genes within the network. (c) Regulatory network analysis highlights CELF1 as a key regulatory node influencing the top 10 interacting genes, particularly HNRNPK, FUS, U2AF2, and HNRNPA2B1

 Topological analysis using CytoHubba identified HNRNPK, HNRNPA2B1, U2AF2, FUS, and CELF1 as hub genes based on degree and betweenness centrality (Figure 3b). These proteins occupied central positions within the network and displayed extensive connectivity with other RBPs. Notably, CELF1 emerged as a key regulatory node interacting with multiple top-ranked hub proteins, including HNRNPK, FUS, U2AF2, and HNRNPA2B1, suggesting a central role in coordinating RNA processing–related interactions within the polyA/β-TERM–associated RBP network (Figure 3c).

###  Generation and Expansion of Stable Recombinant CHO-K1 Cell Pools

 Following puromycin selection, stable CHO-K1 cell pools were successfully generated. The first puromycin-resistant colonies became visible approximately four days after initiation of selection. Cells continued to proliferate under selective pressure, resulting in the formation of stable cell pools over 14 days (Figure 4). At the end of the selection phase, conditioned culture media were collected for downstream analyses, and the established stable cell pools were cryopreserved for further experiments.

**Figure 4:**
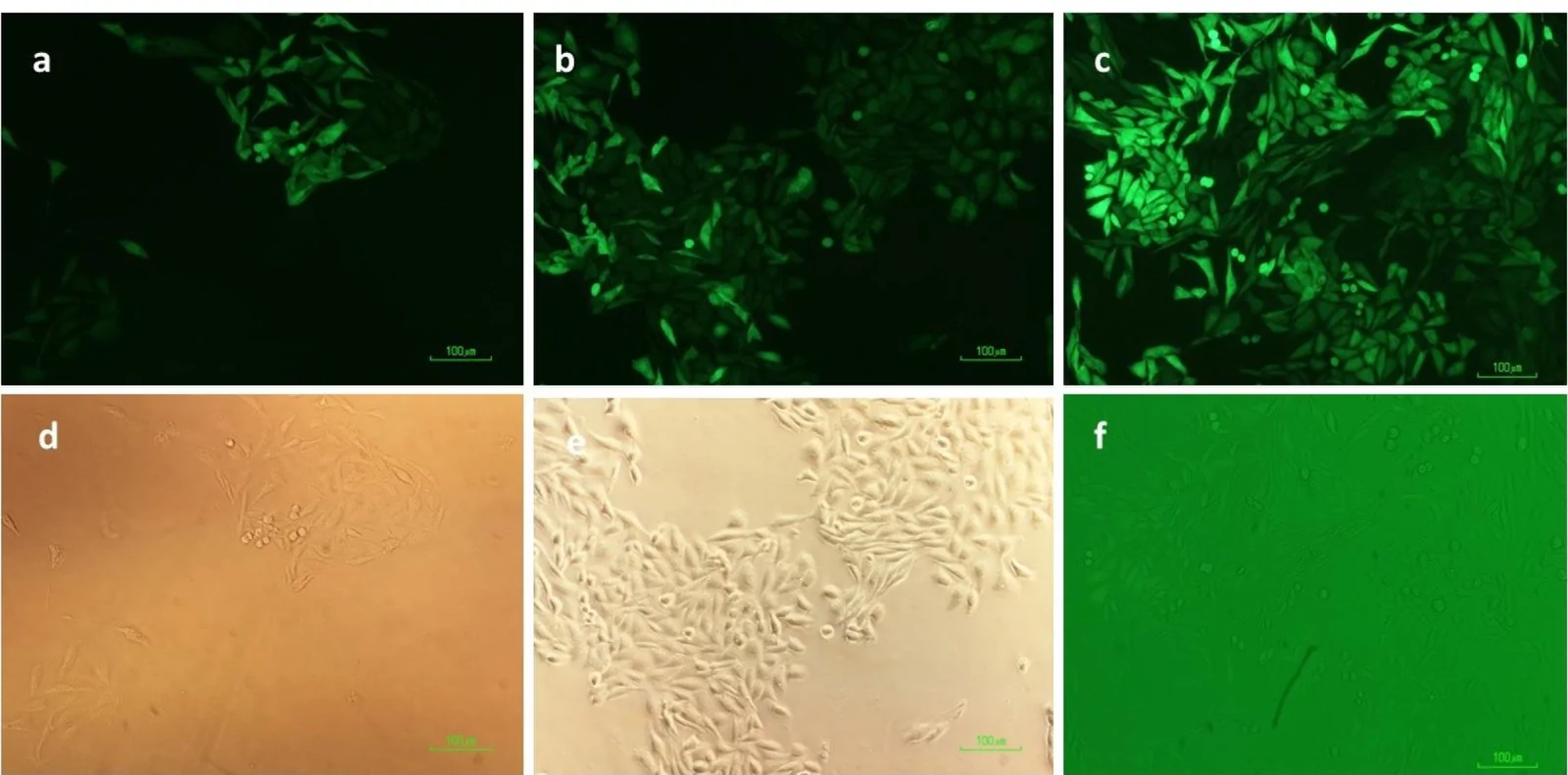
Generation of stable puromycin-selected CHO-K1 cell pools expressing CopGFP. Representative images illustrating the progression of stable CHO-K1 cell pool formation following puromycin selection. After transfection, cells were cultured in selection medium containing puromycin. Puromycin-resistant colonies became visible within 3–5 days and gradually expanded to form stable cell pools over a 14-day period. Fluorescence microscopy images (a–c) and the corresponding bright-field images (d–f) show different stages of colony formation and pool establishment

###  MAR-Mediated Increase in Recombinant EPO Gene Copy Number

 Absolute quantification based on a standard curve was performed on genomic DNA to determine the EPO GCN per cell in the generated stable CHO-K1 cell pools. As shown in (Figure 5a), the pMAR cell pool exhibited the highest gene copy number (15 copies per cell). In comparison, lower copy numbers were observed in the pMT (7.1 copies per cell), pTERM (3.1 copies per cell), and pNMT (1 copy per cell) cell pools.

**Figure 5:**
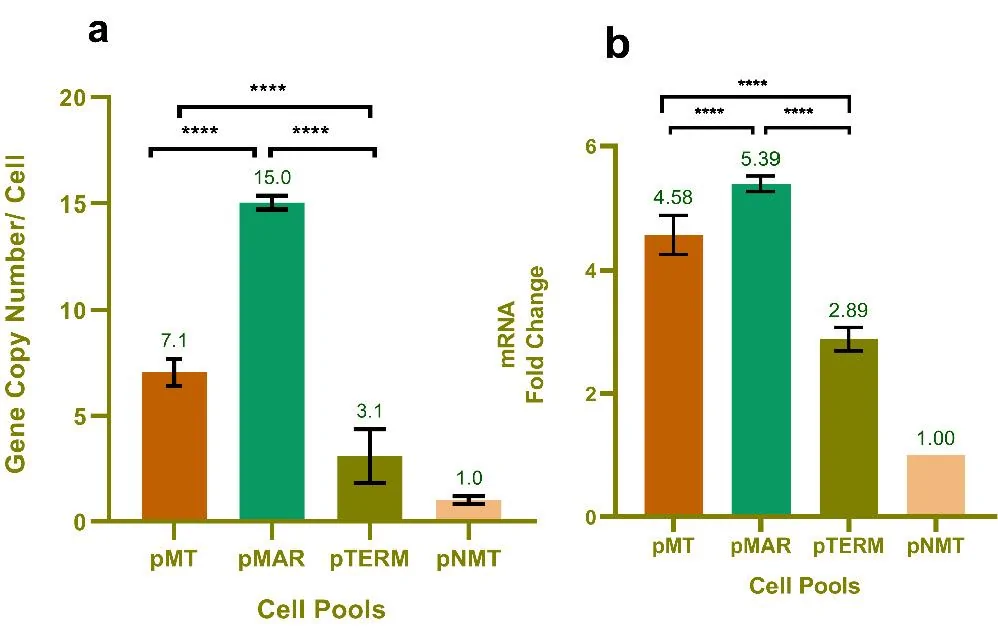
EPO gene copy number and mRNA expression in stable CHO-K1 cell pools. (a) Absolute quantification of EPO gene copy number (GCN) in stable CHO-K1 cell pools determined by qPCR using a plasmid-derived standard curve. The pMAR pool exhibited the highest GCN, followed by pMT, pTERM, and pNMT. (b) Relative EPO mRNA expression determined by quantitative reverse transcription PCR (qRT-PCR). Expression levels were normalized to CHO β-actin and calculated relative to the pNMT pool, which was used as the calibrator. The pMAR pool showed the highest transcript abundance among all stable cell pools. Data are presented as mean ± SD from three independent biological replicates, each analyzed in technical triplicate. Numerical values above the bars indicate mean expression levels. ***P < 0.001, **** *P* < 0.0001

###  pMAR Stable Cell Pools Exhibit the Highest EPO mRNA Expression

 As described previously, relative EPO mRNA expression levels in each stable cell pool were quantified by qRT-PCR. The ΔΔC_T_ method was applied using CHO β-actin as the endogenous reference gene and the pNMT cell pool as the calibrator. Among the generated pools, the pMAR stable cell pool exhibited the highest relative EPO mRNA expression, with a 5.3-fold increase over the other cell pools (Figure 5b).

###  MAR Promotes Robust EPO Protein Production in Stable CHO-K1 Cells 

 Western blotting under reducing conditions was performed on conditioned culture media collected from all stable cell pools. As shown in (Figure 6a), pronounced EPO-specific bands were detected in the pMAR and pMT cell pools, indicating robust protein expression. In contrast, the pTERM pool exhibited only a faint EPO signal, while no detectable band was observed in the pNMT control. Recombinant erythropoietin β (Cinnapoietin; 10,000 IU/0.6 mL) was used as a reference standard for band identification.

**Figure 6:**
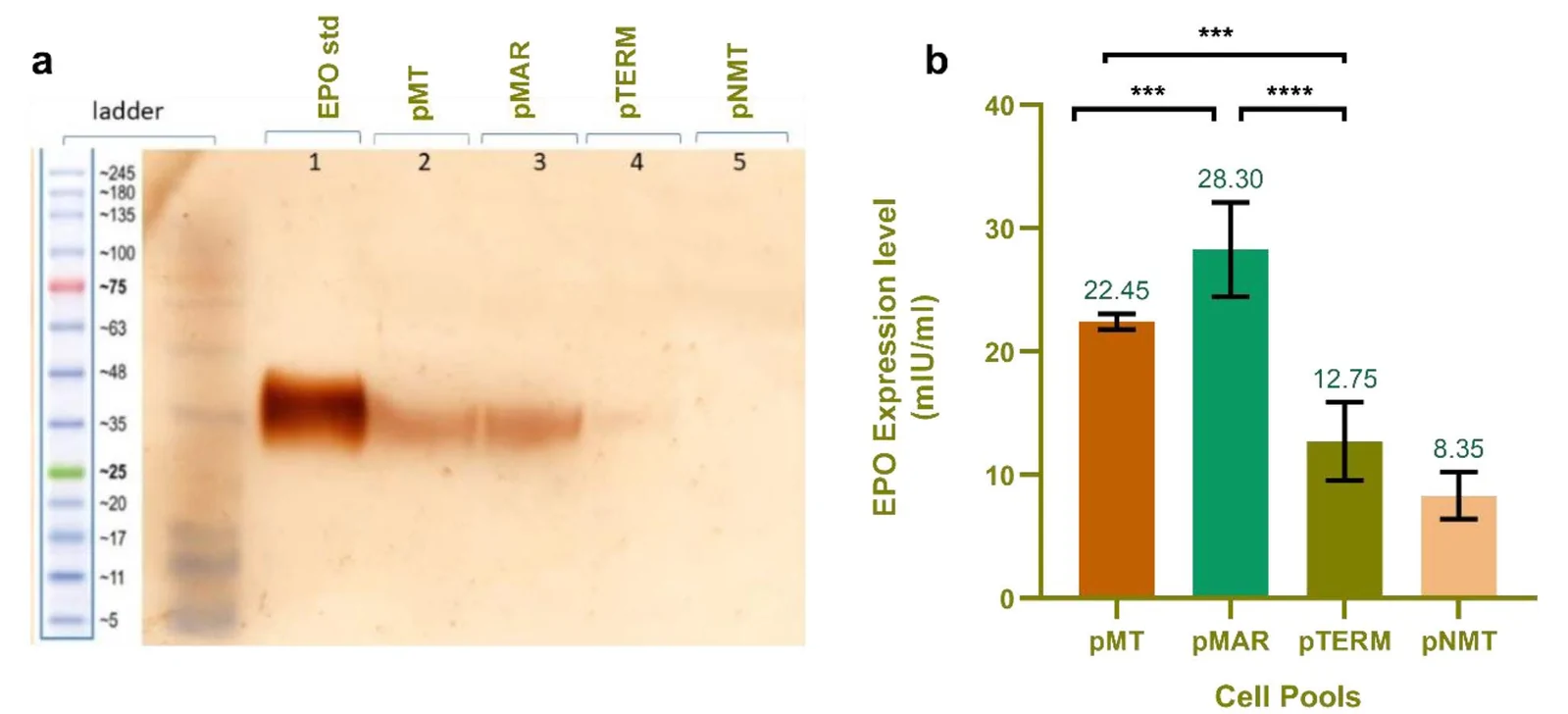
Protein-level characterization of recombinant EPO expression in stable CHO-K1 cell pools. (a) Western blot analysis of recombinant EPO expression in stable CHO-K1 cell pools. Distinct immunoreactive bands corresponding to EPO were detected in the pMAR and pMT pools. A weaker EPO-specific band was observed in the pTERM pool, whereas no detectable band was observed in the pNMT pool. Lane 1, recombinant human EPO standard (erythropoietin β); Lane 2, pMT; Lane 3, pMAR; Lane 4, pTERM; Lane 5, pNMT. Molecular weight markers are indicated in kilodaltons (kDa). (b) Quantification of secreted EPO levels in culture supernatants of stable CHO-K1 cell pools. The pMAR pool exhibited the highest EPO production, followed by pMT, while pTERM and pNMT showed comparatively low secretion levels. Data are presented as mean ± SD from three independent biological replicates, each analyzed in technical triplicate.Numerical values above the bars indicate the mean EPO concentrations measured by ELISA. ***P < 0.001, **** *P* < 0.0001

###  MAR Enhances Secreted EPO Levels in Stable CHO-K1 Cells

 A sandwich ELISA was performed on the culture supernatants of each stable cell pool to quantify secreted EPO. All measurements were conducted using three biological replicates, each analyzed in technical triplicate. As shown in (Figure 6b), the pMAR stable cell pool exhibited the highest level of secreted EPO (28.30 mIU/mL). In contrast, pMT (22.45 mIU/mL) and pTERM (8.25 mIU/mL) displayed markedly lower EPO levels, while pTERM and pNMT exhibited comparably low levels of EPO secretion.

## Discussion

 Cell engineering is a multifactorial process in which transcriptional efficiency, mRNA stability, translational competence, and genomic context collectively determine the productivity of recombinant proteins.^1^ Accordingly, rational vector design that integrates sequence-level optimization with regulatory DNA elements represents a key strategy for improving expression performance in mammalian cell factories such as CHO cells.^2^

 In the present study, EPO expression in CHO cells was investigated using a multilayered engineering strategy combining codon optimization with the incorporation of cis-regulatory elements derived from the human β-globin locus. This approach was designed to establish a transcriptionally permissive and structurally optimized expression cassette, rather than attributing expression outcomes to a single molecular determinant.

 Codon optimization of the EPO coding sequence yielded favorable translational indices (CAI = 0.99 and tAI = 0.98) and an appropriate GC content (67.73%), consistent with efficient expression in mammalian systems.^23,24^ Although no direct experimental comparison with a non-optimized EPO sequence was performed, these parameters support the suitability of the optimized construct as a standardized backbone for evaluating the impact of downstream regulatory elements in CHO cells.

 Beyond codon usage, mRNA secondary structure represents a critical determinant of translational efficiency Optimization of the EPO transcript was therefore extended to minimize the formation of stable inhibitory secondary structures by limiting hairpin-loop spacing and reducing the transcript’s MFE. Lower MFE values have been associated with improved ribosome accessibility and translation initiation efficiency.^23^ Consistent with this principle, computational analysis predicted a stem–loop structure with an MFE of −12 kcal/mol located approximately 12 nucleotides downstream of the start codon, a configuration previously associated with enhanced ribosomal recognition and initiation efficiency.^25^

 To further explore the influence of downstream regulatory elements on RNA folding behavior, secondary structure analyses were performed on the EPO–PolyA and EPO–PolyA–TERM transcripts. While incorporation of the polyadenylation signal increased overall structural complexity, inclusion of the β-globin terminator resulted in a pronounced decrease in free energy, indicating the formation of highly stable secondary structures predominantly confined to the 3′ untranslated region. Comparison of MFE and centroid structures revealed that these changes did not perturb the folding architecture of the EPO coding sequence, suggesting that β-TERM primarily modulates global RNA stability rather than directly influencing translation of the coding region.

 At the genomic level, incorporation of the β-MAR element resulted in a pronounced increase in transgene copy number, EPO mRNA abundance, and secreted protein levels in the pMAR stable cell pool. These findings align with previous reports demonstrating that MAR elements can enhance genomic integration efficiency, stabilize local chromatin architecture, and promote sustained transcription by insulating transgenes from repressive chromatin environments.^7,11^ As noncoding regulatory DNA elements associated with the nuclear matrix, MARs have been proposed to facilitate the formation of transcriptionally permissive chromatin loops, thereby reducing epigenetic silencing and supporting long-term expression stability.^20^ Notably, the β-MAR has been reported to retain functionality irrespective of orientation, provided that the full sequence is preserved.^26^ Previous reviews have also highlighted the importance of chromatin-modifying elements such as MARs and insulators in mitigating position effect–mediated transgene silencing.^27^ Although different elements exhibit context-dependent performance, MARs have consistently been reported to enhance expression stability and reduce variability across integration sites, supporting their selection in this investigation.

 In addition to its structural role, the β-MAR contains multiple predicted transcription factor binding sites, including SATB1, C/EBP, CTCF, and GSH, which may contribute to the enhanced transcriptional activity observed in the pMAR cell pool.^28,29^ Binding of transcription factors and chromatin-associated proteins to these motifs could facilitate recruitment of the transcriptional machinery and promote establishment of an open chromatin configuration. Although direct validation of these interactions was beyond the scope of this study, such mechanisms may contribute to the increased gene copy number and sustained mRNA expression observed in MAR-containing constructs, as suggested by established models and prior literature. Future studies employing chromatin immunoprecipitation (ChIP) or proteomic approaches would be required to directly validate the interaction between MAR-associated transcription factor binding sites and nuclear proteins.

 Despite its pronounced influence on RNA secondary structure, incorporation of β-TERM alone or in combination with β-MAR did not enhance EPO protein production. Although the pTERM cell pool exhibited elevated EPO mRNA levels relative to the pNMT control, this increase was not accompanied by higher protein secretion. This dissociation highlights the multifactorial regulation of gene expression, in which transcriptional output, mRNA stability, and translational efficiency are not necessarily directly correlated. Furthermore, the reduced expression output of constructs combining β-MAR and β-TERM suggests potential regulatory interference between upstream and downstream elements, emphasizing that strong cis-regulatory sequences do not always exert additive or synergistic effects. Accumulating evidence suggests extensive coupling between transcription initiation and termination processes in eukaryotic cells. In particular, physical interactions between promoter-associated initiation factors and 3′ end-associated termination complexes may facilitate the formation of looped gene architectures by spatially associating promoter and terminator regions. Such gene-looping configurations have been implicated in transcriptional termination, reinitiation, maintenance of promoter directionality, and transcriptional memory.^30,31^ These observations raise the possibility that simultaneous incorporation of strong chromatin-associated and termination-associated regulatory elements within the same expression cassette may produce context-dependent regulatory interactions rather than strictly additive effects. It should also be noted that the functional outcome of terminator elements can be highly sensitive to their precise positioning and orientation relative to the polyadenylation signal and transcription unit, parameters that were not systematically varied in this study. Moreover, based on previous studies, the limited enhancing effect of β-TERM observed in the present work may also be influenced by the promoter context used in this vector design. Since promoter–terminator compatibility can substantially affect transcriptional efficiency and transcript processing,^30^ future studies evaluating the combined activity of β-MAR and β-TERM in association with alternative promoters may provide further insight into their context-dependent regulatory behavior. Such interactions may also influence co-transcriptional RNA processing dynamics, including transcriptional pausing, RNA maturation, and recruitment of RNA-binding proteins.^32,33^

 Termination-associated secondary structures and polyadenylation-dependent conformational changes within the transcription complex are known to influence RNA processing, pausing, and degradation kinetics.^13,34^ Each terminator sequence possesses distinct structural and thermodynamic properties, which may differentially affect transcription termination efficiency and transcript fate. In addition, emerging evidence suggests that terminator regions can influence epigenetic regulation by generating noncoding RNAs that recruit transcriptional co-regulator complexes and modulate promoter activity.^35^ Such mechanisms may contribute to the context-dependent behavior of β-TERM observed in the current study. A comprehensive mechanistic understanding of terminator sequence function has not yet been fully established, particularly regarding how terminator positioning and overall transcript architecture influence RNA fate and transcriptional regulation.

 In the present study, incorporation of the β-globin terminator downstream of the polyadenylation signal resulted in increased EPO mRNA abundance without a proportional increase in secreted protein levels. Importantly, elevated transcript abundance does not necessarily indicate improved transcript quality or translational competence. Since qPCR-based mRNA quantification measures total transcript levels without distinguishing between fully processed, translation-competent mRNAs and aberrantly processed, unstable, or non-productive transcripts, the increased mRNA levels observed in β-TERM-containing constructs may partially reflect the accumulation of transcripts that do not efficiently contribute to productive protein synthesis.^34^

 The interaction network analysis provides additional biological context on how the polyA signal and β-TERM may cooperatively regulate post-transcriptional events. The identification of HNRNPK, HNRNPA2B1, U2AF2, FUS, and CELF1 as hub RNA-binding proteins highlights the dominance of factors involved in transcription–RNA processing coupling, mRNA maturation, and stability rather than direct translational enhancement. These RBPs have been extensively reported to localize within transcription factories and to participate in co-transcriptional splicing, RNA packaging, and 3′ end processing, supporting the notion that the β-TERM primarily influences RNA fate at early post-transcriptional stages. Notably, CELF1 emerged as a central regulatory node with extensive connectivity to other hubs, suggesting a coordinating role in integrating RNA processing and stability pathways. These findings indicate that the functional contribution of the β-TERM is more likely mediated through modulation of RNA maturation and regulatory plasticity rather than through direct stimulation of protein synthesis, thereby providing a coherent explanation for its role in stabilizing transgene expression in CHO cells.

 While MAR-based strategies have previously been employed to enhance recombinant protein expression in mammalian systems, most prior studies have focused on the independent evaluation of individual chromatin-regulating elements, such as UCOEs, cHS4 insulators, STAR elements,^8^ or alternative MAR sequences in distinct transgene contexts. ^9,29^ In contrast, the present study provides a systematic comparative evaluation of the individual and combined effects of a well-characterized β-globin MAR and β-globin terminator within a unified codon-optimized EPO expression cassette in stable CHO-K1 cell pools. Importantly, the study integrates comparative analyses at multiple regulatory levels, including transgene copy number, steady-state mRNA abundance, and secreted EPO production across four defined vector configurations. The observation that β-MAR substantially enhances stable transgene expression, whereas β-TERM exhibits context-dependent and non-additive behavior when combined with β-MAR, provides additional insight into the functional compatibility of cis-regulatory elements in mammalian vector design.

 Despite the strengths of the present study, several limitations should be acknowledged. First, mechanistic validation at the molecular level was not performed; therefore, the proposed involvement of β-MAR–associated transcription factor binding sites and potential chromatin remodeling effects is inferred from established models and prior literature rather than supported by direct experimental evidence such as epigenetic profiling or protein–DNA interaction analyses. Second, integration site preferences and chromatin context associated with PiggyBac-mediated transgene insertion were not systematically analyzed; thus locus-dependent effects on transgene expression cannot be fully excluded. Third, this study focused on a single MAR and terminator element; inclusion of additional regulatory elements, such as alternative MARs, UCOEs, STAR elements, or synthetic terminators, could further strengthen comparative interpretation.^8^ Future studies incorporating chromatin-based assays, integration site mapping, and systematic evaluation of upstream and downstream regulatory architectures will be essential to refine further vector design strategies for robust and predictable transgene expression in CHO cells.

## Conclusion

 Collectively, these findings underscore the central role of genomic context and chromatin-level regulation in determining stable recombinant protein expression in CHO cells. While codon optimization and RNA structural tuning provide a favorable molecular framework, the dominant contribution of β-MAR underscores its utility as an effective regulatory element to enhance transgene copy number and transcriptional output. Future optimization strategies may benefit from incorporating alternative MARs, diverse terminator architectures, and systematic integration site analyses using approaches such as PCR-based methods and CRISPR/Cas9-assisted mapping.^36^ Our results further highlight the importance of systematically evaluating combinatorial regulatory architectures, as cis-regulatory elements with individually beneficial properties may not necessarily exhibit additive behavior when incorporated within the same expression cassette. Such combinatorial strategies will be essential for refining vector design principles and maximizing recombinant protein productivity in mammalian cell factories.

## Competing Interests

 The authors declare no conflict of interest. All authors have read and approved the final version of the manuscript.

## Data Availability Statement

 The datasets generated and/or analyzed during the current study are available from the corresponding author upon reasonable request.

## Ethical Approval

 This work was supported by the Golestan University of Medical Sciences (Grant Number 111167; IR.GOUMS.REC.1399.335). This study did not involve human participants or animals and therefore did not require ethical approval.
